# Case report: Application of nonsurgical method in saving transplant renal vein thrombosis caused by acute diarrhea

**DOI:** 10.3389/fmed.2023.1275188

**Published:** 2023-12-15

**Authors:** Liubing Xia, Yongrong Ye, You Luo, Bin Miao, Ning Na

**Affiliations:** Department of Kidney Transplantation, The Third Affiliated Hospital of Sun Yat-sen University, Guangzhou, Guangdong, China

**Keywords:** kidney transplantation, transplant renal vein thrombosis, heparin, acute diarrhea, anticoagulation

## Abstract

Transplant renal vein thrombosis is a rare complication after kidney transplantation, which can seriously threaten graft survival. Though the measures like thrombolytic therapy or operative intervention could be taken to deal with this complication, allograft loss is the most common outcome. Thus, early finding as well as decisive intervention is crucial to saving the graft. Here we present a 46-year-old male patient who underwent kidney transplantation from a cadaveric donor who developed a transplant renal venous thrombosis induced by acute diarrhea more than 1 year after renal transplantation with an initial symptom of sudden anuria and pain in the graft area. Subsequently, serum creatinine levels increased to 810.0 μmol/L. Pelvic CT showed increased vascular density of the transplanted kidney, and contrast-enhanced ultrasound confirmed venous thrombosis. The patient was treated with heparin sodium alone and diuresis gradually resumed. After more than 1 year of follow-up, serum creatinine returned to the baseline level prior to thrombosis. Our case indicates that quick ancillary examination and treatment without hesitation would be indispensable in rescuing allografts with renal vein thrombus. Unfractionated heparin can be recommended as an effective treatment for mid-long-term renal transplantation patients with renal vein thrombosis.

## Introduction

Kidney transplantation has been widely performed around the world as an effective measure to treat patients with end-stage renal disease. However, there are various complications after transplantation, which often threaten the survival of transplanted kidneys and even patients (1). The occurrence of transplant renal vein thrombosis (TRVT) is relatively rare, but it can cause great damage to transplanted kidneys and often leads to graft loss (2).

The causes of TRVT are complex, including donor and receptor risk factors, technical problems, and immunosuppressive factors (3–6). Patients with TRVT often present with pain in the transplanted kidney area, hematuria, sudden anuria or oliguria. Those symptoms are similar to acute rejection and urinary stones and can lead to misdiagnosis and delay in treatment (7). Ultrasound examination may show elevated blood flow resistance at all levels of kidney arteries and even signs retrograde diastolic flow (RDF) (8). Once clinically diagnosed, emergency treatments are often adopted, such as surgical thrombectomy, but the final graft survival rate is extremely low. Here we reported a case of TRVT caused by acute diarrhea and successfully saved the patient’s transplanted kidney by using anticoagulation alone.

## Case presentation

A 46-year-old male patient underwent allogeneic kidney transplantation for “end-stage renal disease” at the Central South Hospital of Wuhan University in July 2020. The immunosuppressive regimen of tacrolimus, methylprednisolone and mycophenolate sodium was administered orally and his serum creatinine recovered to a minimum of 150 μmol/L. About 2 months after the operation, the patient sufferd a rejection and the serum creatinine increased to 400 μmol/L. The creatinine dropped to about 180 μmol/L with a anti-rejection treatment. Eight months prior to this TRVT, the patient had a lung infection and his blood creatinine fluctuated between 200 and 300 μmol/L after recovery.

**Figure 1:**
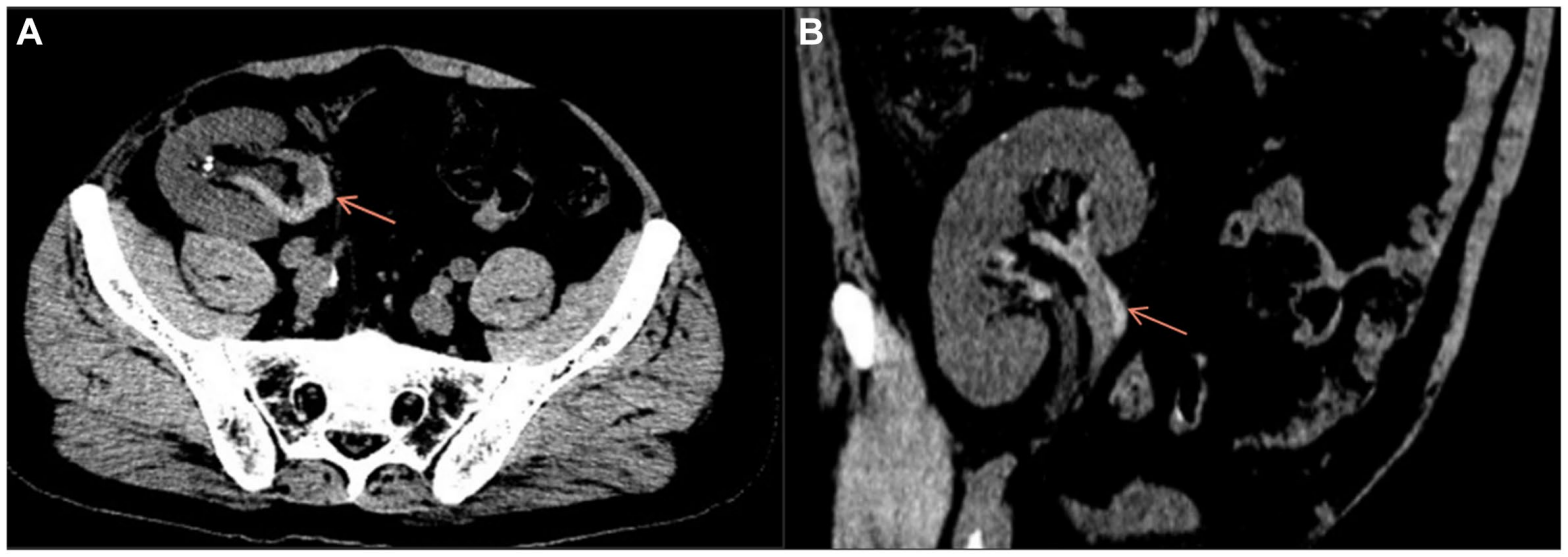
Axial **(A)** and coronal **(B)** pelvic CT scan showed increased vascular density in the transplanted kidney.

In December 2021, the patient had sudden onset of anuria 1 day prior to admission, accompanied by pain in the transplanted kidney area with significant tenderness. The patient’s blood biochemistry in our emergency department indicated that the blood creatinine was 338 μmol/L. Meanwhile, Doppler ultrasound showed that the transplanted kidney was about 111 mm × 57 mm × 22 mm in size, Vmax = 71 cm/s, the graft arterial flow was patent, the resistance index ranging from 0.76 to 0.78 for each level of artery, and no abnormal echogenicity in ureter and bladder was detected. On admission, a pelvic CT scan was performed for the patient to further confirm the diagnosis, which showed multiple small stones in pelvis and calyceal system, mild ureteral dilatation and effusion, multiple exudation of the right iliac fossa involving the peritoneum; the vascular scanning density of transplanted kidney was increased (Figures 1A,B). Asked about the medical history, the patient complained of 3 times of watery diarrhea 2 days prior to admission and 5 times of watery diarrhea again on the same night. He took Montmorillonite Powder for symptomatic treatment on his own, but did not drink in time to supplement the water lost through the digestive tract as he felt no obvious thirst.

The next morning, the tenderness in the graft area was less than before and no obvious swelling of the right lower limbs was observed, but the blood creatinine increased to 830 μmol/L on recheck. Due to persistent anuria, hemodialysis was implemented. Combining the patient’s medical history, physical examination and various tests, urinary stones, acute rejection, and TRVT were all possible causes, but further tests were required to make a diagnosis.

**Figure 2:**
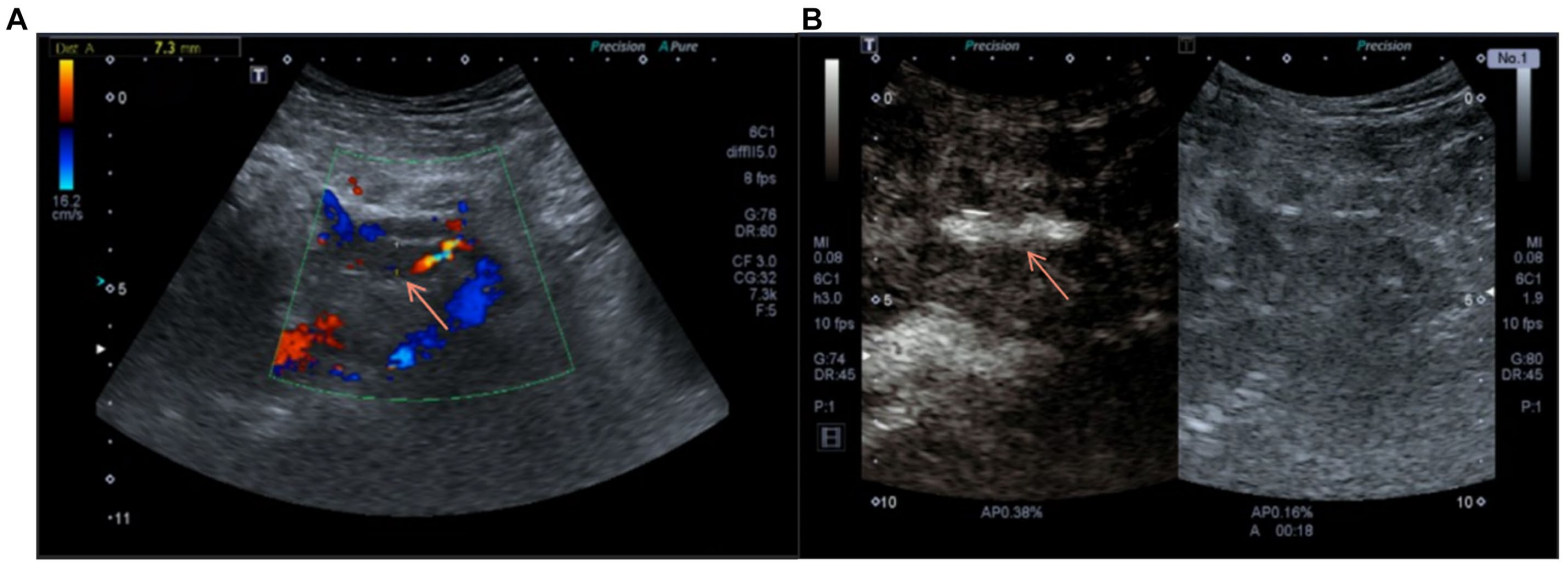
**(A)** Doppler ultrasound on admission suggested the blood flow of the transplanted renal vein was blocked. **(B)** Contrast-enhanced ultrasound before anticoagulation showed the contrast agent passing through the transplanted renal vein.

To further confirm the diagnosis, we performed contrast-enhanced ultrasonography on the patient, which showed that within the transplant renal artery Vmax = 62 cm/s, with a prolonged resistance acceleration time. There is a filling defect with non-enhancing hypoechoic mass of approximately 45 mm × 8 mm in size with in the renal vein lumen, which led to a maximum venous stenosis of >70% (Figures 2A,B). In response to allograft venous thrombosis, we worked with interventional radiologists to assess the condition to formulate a treatment plan using anti-coagulation therapy.

We gave the patient an initial dose of 1,500 U of heparin sodium injection intravenously, followed by 10, 000 U of unfractionated heparin sodium added to saline in a total volume of 50 mL and continuously injected by micropump at a rate of 2–4 mL/h. We monitored blood coagulation every 6–8 h and especially focused on the activated partial thromboplastin time (APTT). The first five days, we maintained APTT at 42.1 ~ 76.2 s. Next four days, heparin was reduced to 8,000 U and continued to be injected by micropump, and the APTT value was maintained at 30.7 ~ 89.6 s.

**Figure 3:**
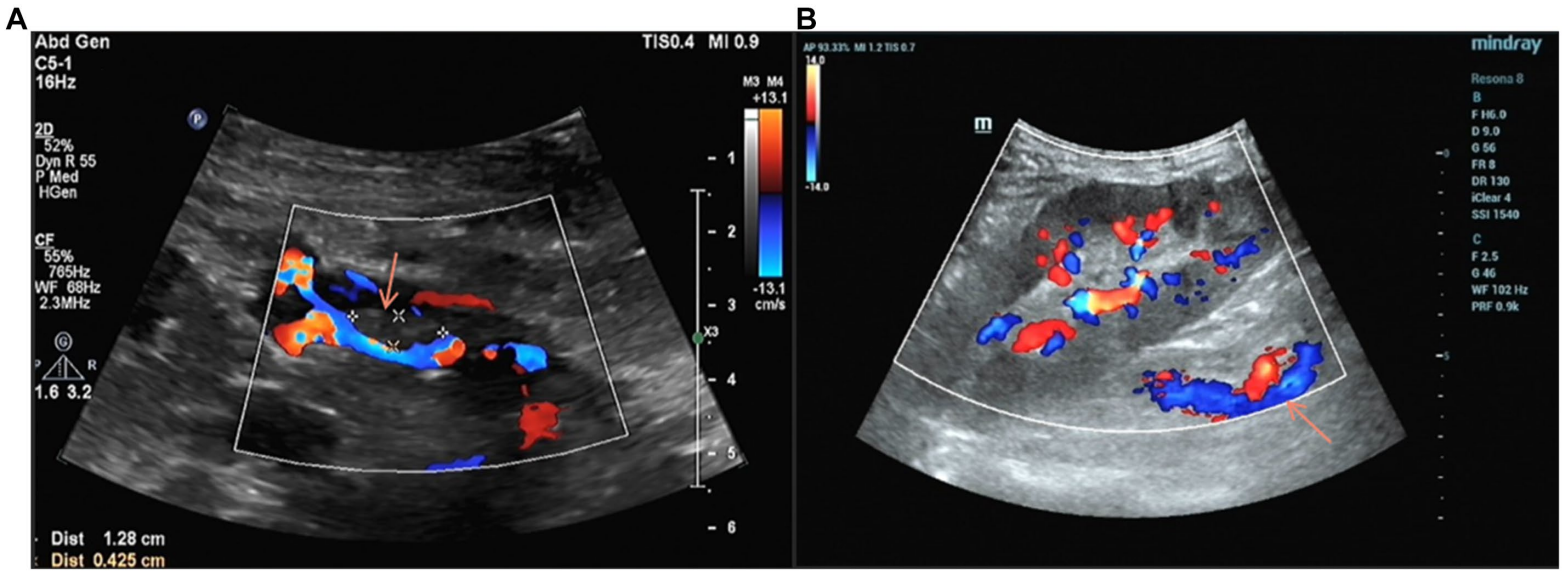
**(A)** Four days after anticoagulation, Doppler ultrasound showed a reduction in the volume of the thrombus within the main trunk of the grafted kidney vein compared to before. **(B)** Anticoagulant therapy for 11 days, the thrombus in the vein lumen of the transplanted kidney disappeared.

On the third day of treatment, the urine output returned to more than 2000 mL/day. Five days after treatment, the ultrasound of the transplanted kidney suggested that the size of the hypoechoic mass in the lumen of the renal vein was approximately 13 mm × 4 mm (Figure 3A). On the 10th day of treatment, a repeat ultrasound of the transplanted kidney showed a clear renal vein wall with continuous echogenicity, the hypoechoic mass in the original lumen was not shown (Figure 3B), and the blood creatinine decreased to 319 μmol/L.

The patient was given warfarin 2.5 mg/d after discharge. The dose was reduced to 1.25 mg/day for 5 months and discontinued after 8 months. During outpatient follow-up of 14 months, blood creatinine maintained between 232 and 396 μmol/L and daily urine output was maintained at approximately 2000 mL per day. A repeat ultrasound of the transplanted kidney showed good venous flow and no significant hypoechoic masses in the lumen.

## Discussion

Kidney transplantation is the most effective treatment for chronic end-stage renal disease. However, with the increasing demand of recipients and use of expanded criteria donors, patients face more complex postoperative complications. Previous reports mostly occur in the perioperative period, the causes include persistent compression by hematoma, abscess or arcuate artery, kinking of the renal vein during placement in the iliac fossa, extension of the iliac vein thrombus into the renal vein, pediatric donor kidney and acute rejection (7). The overall incidence of TRVT is about 0.1–4.2% (9, 10).

Patients with TRVT usually show pain in the transplanted kidney area with tenderness, oliguria, hematuria, and swelling of the lower limb on the transplanted side (9). Our patient presented with watery diarrhea 2 days before admission, followed by sudden onset of right lower abdominal pain with significant tenderness, but no obvious right lower limb swelling. The following day, the patient presented to our emergency department with a sudden onset of anuria. Ultrasound examination only indicated that the arterial resistance index of the transplanted kidney increased at all levels, and the D-dimer was within normal limits. The Pelvic CT and contrast-enhanced ultrasound were performed after admission to confirm the diagnosis of TRVT. Therefore, early and comprehensive examination is particularly important for the diagnosis and follow-up treatment of TRVT.

There have been reports of orthotopic renal vein thrombosis caused by diarrhea especially for newborns or children (11). It was also reported that dehydration could lead to a significant increase in the incidence of deep venous thrombosis after the earthquake (12). However, there are few reports on the formation of allograft renal vein thrombosis after diarrhea in renal transplant patients. On the one hand, acute watery diarrhea can lead to rapid and massive loss of water from the body, reducing the effective circulating blood volume and causing isotonic dehydration (13). Nevertheless, patients may not have obvious symptoms of thirst, fail to replenish water in time, and the blood thickens after concentration, thus promoting thrombosis. On the other hand, the reduction in effective blood volume leads to a redistribution of blood throughout the body to ensure blood supply to vital organs such as the heart and brain, with a corresponding reduction in blood flow to the transplanted kidney and venous blood return is further slowed down, which also contributes to thrombus formation. Therefore, acute watery diarrhea in patients after renal transplantation requires timely water supplementation to prevent the occurrence of TRVT. Additionally, renal sympathetic nerve is critical for the regulation of renal blood flow and glomerular filtration rate (14), and the complete denervation of the transplanted kidney makes the allograft lack of neuroregulation, i.e., in the case of gradual recovery of blood volume, the re-relaxation of the transplanted kidney blood vessels is passive, which might promote the formation of TRVT.

Previous literature has proposed the elements of venous thrombosis: (1) blood flow changes; (2) vessel wall damage; and (3) alterations in the blood (15). Renal transplantation creates a formation of new blood circulation between the external iliac vessels and the transplanted kidney; Long-term chronic rejection after renal transplantation leads to the inevitable micro-damage of the transplanted renal vein, which constitutes the microenvironment of local pro-coagulation of the transplanted renal vein, and also creates conditions for the formation of venous thrombosis of the transplanted kidney. Our patient presented with anuria the day before admission, and it was because of the anuria that the patient further controlled his water intake, resulting in uncorrected dehydration. The patient’s D-dimer increased to 7.03ug / ml on the second day of admission, which indicated a hypercoagulable state of the patient’s blood. Elevated D-dimer often suggests the possible presence of deep vein thrombosis (16), and combined with the patient’s pelvic CT and transplant renal ultrasonography findings, we ultimately made an accurate diagnosis.

The treatment of transplanted venous thrombosis includes surgical thrombectomy, interventional thrombolysis, and intravenous heparin thrombolysis (9). Surgical treatment is the main treatment after perioperative venous thrombosis (17, 18). Thrombolysis with urokinase or streptokinase under interventional conditions can apply to transplanted renal vein thrombosis combined with ipsilateral lower extremity deep vein thrombosis (19), but the inferior vena cava filter needs to be placed in advance, and a large amount of contrast agent is required during the thrombolysis process, which will undoubtedly have a negative impact on the recovery of transplanted kidney function (20). There is a previously reported case of allograft renal vein thrombosis associated with deep vein thrombosis with no apparent cause, which was successfully treated with plain heparin sodium in combination with elastic compression stockings (21). Considering that the patient had undergone surgery for more than 2 years without hemorrhagic tendency and Doppler ultrasound showed the lumen of the transplanted renal vein was not completely obstructed, we gave the patient a regimen of intravenous heparin anticoagulation and dynamic monitoring of the APTT to adjust the dose of medication.

The incidence of venous thrombosis in the transplanted kidney is on the rise with the widespread introduction of kidney transplantation. Diarrhea as a trigger for TRVT formation is rarely reported, especially acute watery diarrhea that can lead to massive water loss and blood concentration in a short period of time is of concern. Early recognition and timely targeted therapy is equally critical to saving the transplanted kidney. According to our experience, unfractionated heparin anticoagulation can be used as an effective method for the treatment of incomplete graft renal vein obstruction caused by TRVT in mid-long term renal transplant patients, but only after a comprehensive assessment of bleeding risk.

## Data availability statement

The original contributions presented in the study are included in the article/supplementary material, further inquiries can be directed to the corresponding author.

## Ethics statement

The studies involving humans were approved by Ethics Committee of the Third Affiliated Hospital of Sun Yat-sen University. The studies were conducted in accordance with the local legislation and institutional requirements. The participants provided their written informed consent to participate in this study. Written informed consent was obtained from the individual(s) for the publication of any potentially identifiable images or data included in this article.

## Author contributions

LX: Data curation, Writing – original draft, Writing – review & editing. YY: Writing – original draft, Writing – review & editing. YL: Data curation, Writing – review & editing. BM: Supervision, Writing – review & editing. NN: Supervision, Writing – review & editing.
